# Nasal Delivery of High Molecular Weight Drugs

**DOI:** 10.3390/molecules14093754

**Published:** 2009-09-23

**Authors:** Yıldız Ozsoy, Sevgi Gungor, Erdal Cevher

**Affiliations:** Istanbul University, Faculty of Pharmacy, Department of Pharmaceutical Technology, 34116-Universite, Istanbul, Turkey

**Keywords:** macromolecular drugs, nasal delivery, vaccine, peptide, protein

## Abstract

Nasal drug delivery may be used for either local or systemic effects. Low molecular weight drugs with are rapidly absorbed through nasal mucosa. The main reasons for this are the high permeability, fairly wide absorption area, porous and thin endothelial basement membrane of the nasal epithelium. Despite the many advantages of the nasal route, limitations such as the high molecular weight (HMW) of drugs may impede drug absorption through the nasal mucosa. Recent studies have focused particularly on the nasal application of HMW therapeutic agents such as peptide-protein drugs and vaccines intended for systemic effects. Due to their hydrophilic structure, the nasal bioavailability of peptide and protein drugs is normally less than 1%. Besides their weak mucosal membrane permeability and enzymatic degradation in nasal mucosa, these drugs are rapidly cleared from the nasal cavity after administration because of mucociliary clearance. There are many approaches for increasing the residence time of drug formulations in the nasal cavity resulting in enhanced drug absorption. In this review article, nasal route and transport mechanisms across the nasal mucosa will be briefly presented. In the second part, current studies regarding the nasal application of macromolecular drugs and vaccines with nano- and micro-particulate carrier systems will be summarised.

## Introduction

In recent years the nasal route has gained importance as a non-invasive drug application route that offers many advantages for the introduction of drugs into systemic circulation. Its major advantage is the rapid absorption of drugs and therefore quick onset of their effect. In addition, it has the advantage of avoiding the hepatic first-pass effect. It is not however without disadvantages, the best known of which, particularly for macromolecular drugs, are its enzymatic barriers and the low permeability of the nasal epithelia.

Most of the drugs used in the treatment of local inflammation of the nasal mucosa and cavity have very low molecular weights. Among these drugs are steroids and chromaline derivatives. Among the drugs applied via nasal route for systemic effects are small polypeptides such as vasopressin and oxytoxin.

Recent studies, however, have focused on systemic application of other therapeutic agents such as drugs with high molecular weight and those intended for immunization. These drugs cannot be absorbed via an oral route and their bioavailability when they are applied nasally is very low. The bioavailability of the peptide and proteins is inversely proportional with their molecular weight and number of amino acids. In other words, the absorption of drugs via nasal mucosa decreases as the molecular weight increases. Absorption is particularly low for the drugs applied as simple aqueous solutions, so several strategies have been developed to improve the nasal bioavailability of drugs and to apply macromolecular drugs and antigens nasally. Among the most popular approaches are the inclusion of enhancers in the formulations and the preparation of nano- or micro-particulate systems with various polymers.

This review article provides general information on the nasal passage routes of drugs and improving the passage through the nasal mucosa, and an overview of some recent studies during the last two decades on nasal applicaton of enhancer-included formulations of drugs with macro-molecular structure and nano- or micro-particulate carrier systems.

## Nasal Mucosa and Enhancing Drug Passage through the Nasal Route

### Nasal route

Compared to other biological membranes the nasal mucosa is a rather porous and thin endothelial basal membrane. It also has a rapid blood flow, with a highly vascularized epithelial layer and a vast absorption area (150 cm^2^) with microvilli in epithelial cells. Due to these characteristics, it offers many advantages such as fast absorption of drugs, rapid action and low risk of overdose [1,2,3]. Among the major disadvantages of the nasal route are the limited application volume (25-250 µL), the difficulty of high molecular weight drugs (>1,000 Da) to pass through the nasal mucosa, the presence of pathological conditions, mucocilliary drug clearance, enzymatic barriers and irritation of the nasal mucosa [2,4].

The nasal route consists of three functional areas, namely the *vestibular, respiratory and olfactory* areas [1,3,4,5]. Particularly the *respiratory area*, with its rich vascularity and vast surface area, is where the drugs are absorbed to the greatest extent. Retention of nasally-applied drugs in this area is subject to various factors such as particle size of the drug, density, shape and hygroscopicity, respiration and the presence of pathologic conditions in the nasal cavity. While particles larger than 10 µm can be accumulated in the respiratory area via respiration, those smaller than 5 µm are inhaled and reach the lungs, and those smaller than 0.5 µm are exhaled [6,7].

The nasal respiratory mucosa is covered with mucus. The mucus is 5 µm thick and has a viscous gel on the upper part and an aqueous sol layer on the lower part [3]. The mucosal secretion contains 95% water, 2% mucin, 1% salts, 1% albumin, immunoglobulin, lysozyme, lactoferrin and other proteins and 1% lipids. Nasal mucus also contains IgA, IgE and IgG. Nasal epithelia are covered with a new mucus layer approximately every 10 minutes [8]. All components in the air inhaled from outside via the respiratory channel adhere to the mucus in the nasal cavity or are dissolved in the mucus and pushed to the nasopharynx to be thrown into to the gastrointestinal channel. The clearance of the mucus and the components that are adsorbed/dissolved to the gastrointestinal tract is named as *“mucocilliary clearance”* [1]. Epithelial cells have thin, hair-like structures, or *cilia*, on the surface. Every cell has approximately 300 cilia. The mucus layer makes a fluctuating movement together with the underlying cilia. Nasally-applied drugs are cleared from the nose within a half-life of approximately 21 minutes with this movement [5].

### Passage routes of drugs via nasal mucosa

**Figure 1:**
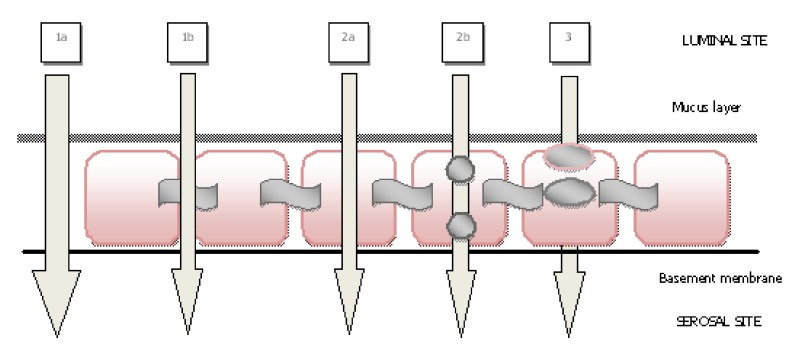
(1) Paracellular route (1a) intercellular spaces, (1b) tight junctions, (2) transcellular route (2a) passive diffusion, (2b) active transport, (3) transcytosis (modified from Ref. [9]).

The passage of drugs via the nasal mucosa is mainly achieved in three ways, which are *paracellular, transcellular* and *transcytotic* (Figure 1) [9,10]. The first route is the *paracellular transport*, which is associated with the intercellular spaces and tight junctions. Paracellular transport is an important route particularly for absorption of peptides and proteins, so it has been reported that the paracellular route should be reversibly opened to enhance nasal absorption of peptides, and mucosal absorption increases due to the hydrophilic characteristic of drugs [11,12]. The second passage route is the *transcellular route* which is achieved with passive diffusion or active transport mechanism. It is important in absorption of lipophilic molecules or the molecules that are recognized by the membrane (active carrier transport) [1,4,9]. The third passage route is *transcytosis*. Here, the particle is taken into a vesicle and transferred to the cell. Finally, it is accumulated in the interstitial space [13,14].

### Improving nasal mucosa transport 

Among the factors affecting the absorption of drugs through the nasal mucosa and their bioavailability are the physico-chemical properties (ionization, lipophilicity, etc.) of the drug, surface charge and hydrophobicity as well as the molecular size, which is the most important factor [13]. Hydrophilic drugs with low molecular weight are absorbed via the nasal mucosa at a rate that is almost comparable to intravenous application [15,16]. Nevertheless, the nasal mucosa is an obstacle for the passage of large molecules, particularly for those above 1,000 Da in size [13], and therefore, the nasal bioavailability of hydrophilic peptides and proteins is usually less than 1% [17]. This low bioavailability of these drugs is associated with the weak mucosal membrane permeability and the presence of proteolitic enzymatic activity in the nasal mucosa. Another reason is the mucocilliary clearance, through which they are rapidly removed from the nasal area [18]. To overcome these disadvantages, different approaches have been proposed to enhance nasal absorption of molecules with peptide and protein structures [19,20,21], which can be summarized as follows:

(a) Modification of the chemical structure of the peptide to increase metabolic stability and/or membrane permeability [19].
(b) There are oxidative, conjugative enzymes, and exopeptidases and endopeptidases in the nasal cavity [22]. This vast diversity of enzymes leads to a “pseudo first pass effect” which hinders the absorption of protein peptide drugs. The drug may be applied with enzyme inhibitors to protect them from the activity of these enzymes in the mucosa [21].
(c) Inclusion of absorption enhancers (such as bile salts and surfactants, fusidic acid derivates, phosphatidylcholines and cyclodextrines) to enhance the passage of drugs with polar structure through nasal mucosa [23,24],
(d) Development of novel formulations including drug carrier systems (liposomes, lipid emulsions, niosomes, nano- and micro-particles) [25,26,27,28,29,30].

One of the preferred approaches among these is the nano- and micro-particulate systems, which are prepared primarily with mucoadhesive polymers to provide sufficient retention time of the drug for absorption in the nasal cavity. Particulate systems facilitate the passage of peptide and protein structured drugs through the nasal mucosa and protect them from enzymatic activity by increasing the retention time of the drug in the nasal cavity, establishing tight contact between the nasal mucosa and the drug, providing localization of the drug at high concentrations, and opening the tight junctions between the epithelial cells.

### Nano-/ micro-particulate systems

Nano- and micro-particles are matrix systems where the drug is dispersed in the polymeric material. These particles are produced with different encapsulation methods, including spray-drying, solvent-evaporation and phase separation [31,32,33]. In nano- and micro-particulate carrier systems, the drug is loaded via either incorporation with the system or its adsorption on the particulate system. Drug is released from the particles through certain mechanisms, which are: (a) release from the particle surface, (b) diffuson of the drug from the swollen polymer matrix, or (c) drug release through the erosion of polymers [34].

The nano- and micro-particulate systems, which are prepared for nasal systemic effect of macromolecules, generally use degradable starch, dextran, chitosan, microcrystalline cellulose (MCC), hydroxypropyl cellulose (HPC), hydroxypropyl methylcellulose (HPMC), carbomer, and wax-like maize starch, gelatin polymers [35].

The mucoadhesive properties of these systems are an important factor in their retention and action in the nasal mucosa. Chitosan, which is a positively charged polymer with a strong mucoadhesive property, is frequently used in nasal application of macromolecules [36,37]. Mucoadhesion is achieved by the ionic interaction of positively charged amine groups of d-glucosamine units of chitosan with negatively charged sialic acid groups of musin or other negatively charged groups of the mucosal membrane [38]. The effect of chitosan that enhances penetration has been associated with its mucoadhesive property as well as its ability to transiently open the tight junctions in the nasal mucosa. It has been reported that chitosan does not lead to any histological changes in the nasal mucosa [39,40,41].

Among the polymers used particularly in nasal application of antigens are poly(l-lactic acid) (PLA) and poly(d,l-lactide-co-glycolide) (PLGA). These polymers have been approved by the Food and Drug Administration (FDA) and they are transformed into lactic and/or glycolic acid in the body. Mucoadhesive polymers like alginate and Sephadex^®^, poly(vinyl alcohol) and chitosan are used together to increase mucoadhesiveness of PLA and PLGA polymers [42,43,44].

## Macromolecular Drugs

The number of macromolecular drugs that are applied nasally is increasing gradually. Those drugs that are approved by the FDA and available in the drug market are listed in Table 1. The molecular weight of these drugs is between 1,000-3,400 Da. Their nasal bioavailability is approximately 10%. However, these drugs are intended for non-parenteral application considering their clinical advantage, practical application and cost of development.

The nasal route, which is particularly preferred for patient compliance purposes, has been widely investigated for noninvasive delivery of high molecular weight drugs. The primary macromolecular drug for which development for nasal application is planned is insulin. Among the commercially available drugs which still under investigation, are desmopressin, salmon calcitonin and a luteinizing hormon releasing hormon (LHRH) agonists. Besides, there are growth hormone (hGH), heparin, α-cobrotoxin, exenatide, glucagon, hirudin and octreotide. Some of the studies with enhancers and particulate systems to enhance nasal absorption of these macromolecular drugs are included in this compilation.

### Insulin

Insulin is a peptide hormone consisting of 51 amino acids whose molecular weight is 6 kDa. Since its first discovery in 1922 to the present, numerous non-invasive routes have been tried to improve insulin treatment and the quality of life of the patients suffering from *diabetes mellitus*. Until very recently insulin had only been applied by subcutaneous routes. In 2006, another preparation (Exubera^®^, Pfizer), which is applied by the pulmonary route has been introduced to the drug market after approval by the FDA. However, the company decided to withdraw the product from the market in October 2007 as sales of Exubera had proven disappointing, with the product failing to gain acceptance from either patients or clinicians.

The nasal route for insulin delivery has been one of the mostly studied alternative routes due to its advantages. Some of these studies are summarized in Table 2. As seen in the table, since insulin has a high molecular weight, numerous formulation approaches have been tried to improve its absorption through the nasal mucosa. Among these approaches are carrier systems such as powders [46,48,50,51,54], microspheres [45,47,49,53,55,57,58,63], and nanoparticles [52,56,59,60,61,62], most of which are prepared with mucoadhesive polymers. Another approach is the use of permeation enhancers with different structures to overcome the barrier characteristic of the nasal mucosa [45,49,52,58,59,61,62,63].

**Figure 2:**
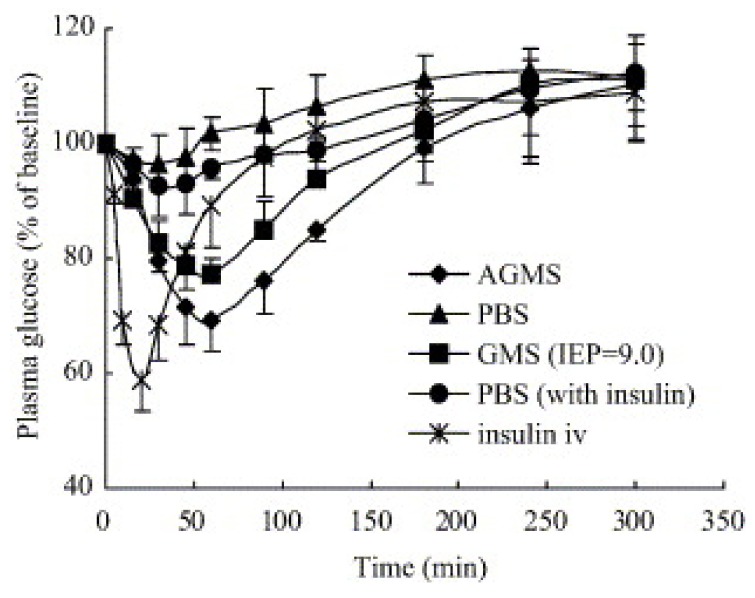
Changes of plasma glucose levels after intravenous administration of insulin solution and intranasal administration of insulin-incorporated gelatin (GMS) and aminated gelatin microspheres (AGMS) in dry powder forms. The dose of insulin was 0.5 IU/kg for intravenous route and 5 IU/kg for intranasal route (PBS-phosphate buffer saline). Each point represents mean ± SD (*n* = 4–5) [reprinted with permission from Ref. [64], copyright Elsevier (2006)].

In one of these studies, aminated gelatin microspheres were prepared as nasal drug delivery system for peptide drugs [63]. *In vitro* studies have demonstrated that these microspheres had a significantly slower release than the native gelatine microspheres. The microspheres prepared were applied nasally to rats in the form of powders and suspensions. The effect of the aminated gelatin microspheres on enhancing absorption was found to be significantly higher in powder formulations (Figure 2). 

These microspheres led to a significant fall in plasma glucose levels. The suspension, on the other hand, did not lead to a significant hypoglycemic effect. The absolute bioavailability after nasal application of both microsphere formulations containing 5 IU insulin was found significantly higher in powder form than suspension form. It has been demonstrated that the powder microspheres draw water from the nasal mucosa due to their hydrogel nature, consequently leading to transient dehydratation of epithelial membrane and opening of tight junctions. The aminated gelatin microspheres, due to their positive charge and mucoadhesive characteristic, are suggested as a novel carrier system for macromolecules.

**Figure 3:**
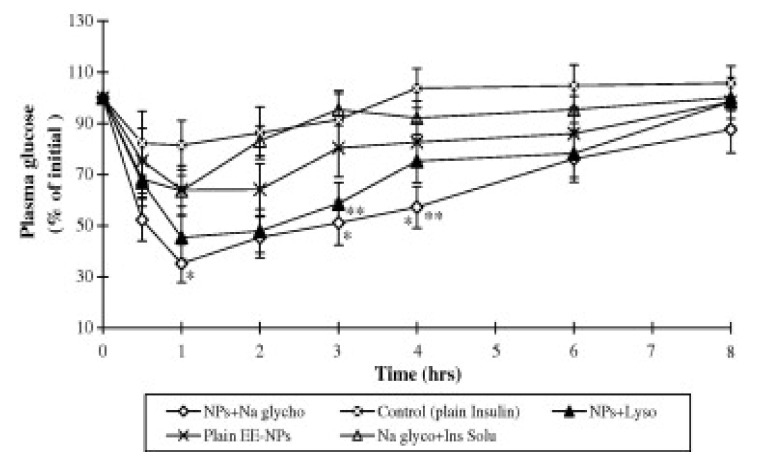
Comparative hypoglycemic effects of EE–NPs (crosslinked with epichlorohydin/prepared emulsion method nanoparticles) in the presence of Na glycocholate and lysophosphatidylcholine after nasal administration to STZ (streotozotocin) induced diabetic rats (mean ± SE, *n* = 5) [reprinted with permission from Ref. [52], copyright Elsevier (2008)].

^*^ Significantly higher effect (*P* < 0.05) when Na glycocholate was co-administered compared to lysophosphatidylcholine and plain NPs without permeation enhancers. ^**^P < 0.05 compared to plain NPs without permeation enhancers.

In another study, insulin nanoparticles were prepared by cross-linking epichlorohydrin with starch in the presence of a permeation enhancer such as sodium glycocholate or lysophosphatidylcholine [52]. These particles were nasally applied to rats. The particles containing sodium glycocholate increased the plasma insulin levels significantly. Besides, these particles produced a higher hypoglycemic effect when compared to nanoparticles that contain lysophosphatidylcholine (Figure 3).

**Figure 4:**
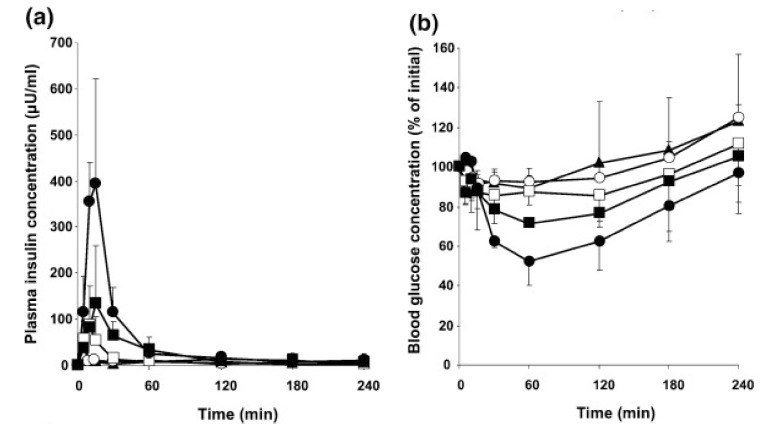
Plasma insulin (a) and blood glucose (b) concentration vs. time profiles following nasal administration of insulin (10 IU/kg) with different CPPs (0.5 mM). Each data point represents the mean ± SEM (*n* = 3). Key: (▲) insulin; (○) L-R8 (specific L-penetratin); (□) D-R8 (specific D-penetratin); (●) l-penetratin; (■) d-penetratin [reprinted with permission from Ref. [64], copyright Elsevier (2009)].

The effect of cell-penetrating peptides (CPPs) was evaluated on the nasal absorption of insulin [64]. CPPs have dramatically increased the nasal absorption of insulin. l-Penetratin was found to be the most effective enhancer of insulin absorption compare to other CPPs. However, increasing the d-penetratin concentration let to a decrease in the efficiency of nasal insulin absorption (Figure 4).

In conclusion, the particulate systems and enhancers have generally improved the transport of insulin through the nasal mucosa. These findings suggest that nasal formulations of insulin could be introduced to the drug market in the future.

### Desmopressin

Desmopressin, which is a vasopressin analog with a molecular weight of 1,069 Da, is commercially available in several dosage forms, including oral tablets (Minirin^®^ tablet), fast-dissolving oral tablets (Minirin^®^ freeze-dried tablet) and a nasal liquid (Minirin^®^ Nasal Spray). The absorption rates of these three dosage forms are different from each other. Nasal spray has the highest absorption capacity (3-5%). Sublingual freeze-dried tablet and the oral tablet have absorption rates of 0.25% and 0.08-0.16% respectively. Although the nasal spray has a better absorption than the other dosage forms, its bioavailability varies. This variability is explained with the difference in the amount of liquid remaining in the nasal cavity [65].

There are some studies in progress to improve the bioavailability of desmopressin. In these studies, the efficacy of mucoadhesive microspheres have been evaluated [66]. In another study conducted most recently, a powder formulation has been prepared using sodium starch glycolate polymer [67]. In this study, the desmopressin powder formulation was administered nasally to healthy volunteers. Desmopressin was uniformly distributed in the nasal powder formulation (CV is 2.4%), sublingual tablets had also homogenous desmopressin content (CV 1.25%). The pharmacokinetic parameters of nasal powder and sublingual tablet formulations have been compared to those of commercial nasal liquid spray. The nasal powder formulation has demonstrated a three-fold increase in absorption when compared to the liquid spray. A lower variability (CV values are 32.0 and 29.8% for AUC _0-12 h_ and C_max_, respectively) has been observed in the nasal absorption of desmopressin with nasal powder formulation. The tablet formulation has not improved the uptake of desmopressin due to its poor sublingual disintegration. Besides, this powder formulation has been suggested as a promising carrier system because of its potential for an easier scale-up process.

### Salmon calcitonin

Salmon calcitonin (sCT) is a peptide hormone consisting of 32 amino acids with a molecular weight of 3.4 kDa. It has been approved by the FDA as a nasal spray (Miacalcin^®^, Novartis) for treatment of postmenopausal osteoporosis and Paget’s disease of bones. The nasal absorption of salmon calcitonin is very low (3%) and therefore formulation development studies are still in progress. Some of these studies are summarized below.

In a study by Morimoto *et al*. [68] sCT was applied nasally to rats in positively and negatively charged gelatin microspheres. The positively charged microspheres demonstrated a better ability to adhere to the mucosa, and gelatin microspheres protected the sCT from enzymatic degradation. The hypocalcemic effect obtained with the microspheres was found significantly higher than that of sCT solution. In conclusion, it has been demonstrated that gelatin microspheres are a beneficial drug carrier for nasal application of peptide drugs.

A sCT spray was prepared with chitosan, which is another mucoadhesive polymer, and applied nasally to sheep [69]. The nasal absorption was compared to that of the commercially available spray and the nasal solution which does not include chitosan. The average relative bioavailability of sCT from chitosan solution was improved two-fold compared with Miacalcin^®^ nasal spray and three-fold compared with sCT control solution. It has been suggested that the drug can be applied i.n. at lower doses and the treatment efficiency can be improved by i.n. application. 

The hypocalcemic effect of sCT conjugated with polyethylene glycol (PEG) at different molecular weights has been evaluated in rats. It has been demonstrated that the i.n. absorption of the PEG conjugates prepared is inversely proportional with the molecular weight of PEG. Besides, the highest effect was achieved with the conjugates prepared with 2 kDa [70].

Permeation enhancers are frequently used in improving the i.n. absorption of sCT. Among these enhancers sodium tauro-24,25-dihydrofusidate [71], sodium glycolate and dihydrofusinate [72], *N*-acetyl-l-cysteine (NAC) [73,74], polyoxyethylene-C25-lauryl ether [75] are used.

**Figure 5:**
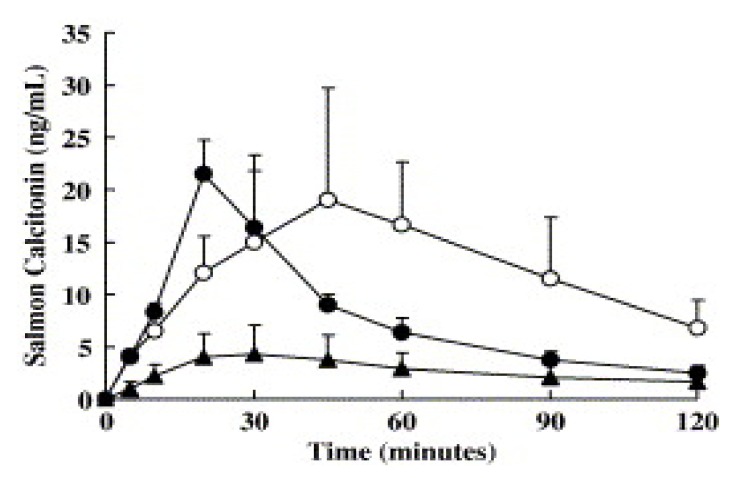
Comparison of plasma concentration–time profiles following nasal administration of liquid and powder formulations, and subcutaneous administration (○) of 0.3 mg of sCT in dogs. ▲; Formulation-L (sCT in saline), ●; Formulation-PN (powder formulation with NAC and ethylcellulose). Data represent mean plasma concentrations of sCT ± S.D. (*n* = 4) [reprinted with permission from Ref. [74], copyright Elsevier (2006)].

Matsuyama *et al*. [74] have evaluated the enhancer effect of NAC *in vivo*. Powder formulations of sCT with ethylcellulose were prepared with and without NAC. These formulations were applied nasally to dogs. The bioavailability of the powder formulation containing NAC, the powder formulation without penetration enhancer and solution applied via subcutaneous route were calculated as 24.9%, 8.7% and 50.9%, respectively. The plasma concentration-time profiles are shown in Figure 5. Besides, it has been reported that the formulation containing NAC achieves the maximum plasma concentration faster than the subcutaneous route. It has been demonstrated that the enhancer used decreases the viscosity of the mucus, leads to changes in epithelial membrane with its surfactant effect and consequently enhances permeability.

### LHRH-agonists

Buserelin, nafarelin and leuprolide, which are luteinizing hormone releasing (LHRH) agonists, are nasally used in treatment (prostatic cancer, endometriosis, central precocious puberty, etc.). These peptides are small peptide hormones that contain 9-10 amino acids. Nasal preparations have a low bioavailability (i.e., buserelin 3%, nafarelin 2.8%) [19]. There are certain studies in literature which have tried to improve the nasal absorption of leuprolide acetate [76], buserelin [77] and goserelin [78] among these peptides.

### Human growth hormone 

Human growth hormone (hGH) is a protein with a molecular weight of approximately 22 kDa that contains 191 amino acids. It is used in the treatment of hormone deficiency, Turner’s syndrome and chronic kidney failure. Its preparations for injection are available in the market. Like other protein and peptide drugs, application of hGH via non-invasive routes has also been studied. It has been estimated that among these routes, the nasal route can better simulate the normal endogenous pulsatile hGH secretion pattern when compared to the subcutaneous injection [1].

The effect of several permeation enhancers on improving nasal bioavailability of hGH was studied. In these studies, lysophosphatidylcholine [79], didecanoyl-l-α-phosphatidylcholine [80,81], sodium tauro-24,25-dihydrofusidate [82] and α-CD [81] have been used as permeation enhancers. In another study, microparticulate systems of hGH were prepared with the thiomer (polycarbophil-cysteine polymer). Besides, gluthatione was added to the microparticles as permeation mediator. The microparticles containing gluthatione increased the relative bioavailability approximately by three folds when compared to the microparticles that did not contain glutathione. It has been stressed that this polymeric system and glutathione can be used as a new system in nasal application of hGH and other peptide drugs [83].

### Heparin

Heparin has a molecular weight of 6-30 kDa. Heparin has been used as an anticoagulant in standard treatment of deep vein thrombosis by a parenteral route for almost 40-45 years. Its unfractioned (UFH) and fractioned injectable preparations with low molecular weight (LMWH) are available in the market. There are certain studies which are still in-progress for non-parenteral application of heparin to improve patient compliance and to minimize the adverse clinical effects. Different strategies have been studied in i.n. application of heparin. The most recent studies concerning this issue are summarized in Table 3. The systems included in this table have been demonstrated to release heparin effectively not only for systemic thrombosis treatment, but also for localized conditions (e.g., allergies [84,85]).

It is seen that these studies also use several permeation enhancers to improve nasal absorption of drugs with a high molecular weight [87,88,89,90]. Besides, nanoparticles [59] and microspheres [86] have been used to improve nasal absorption of heparin.

**Figure 6:**
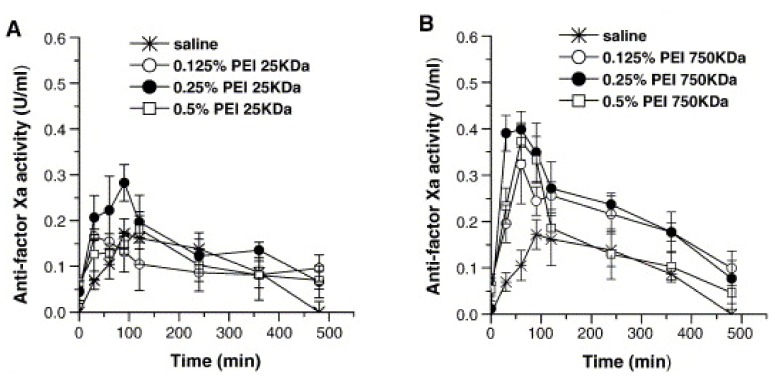

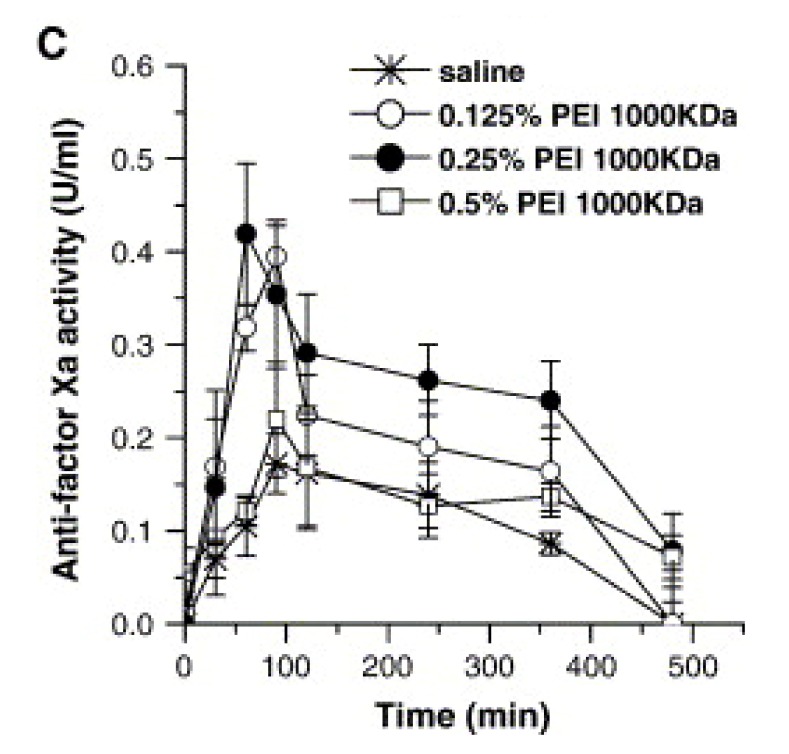
Changes in anti-factor Xa activity after nasal administration of enoxaparin formulated in saline or in the presence of different concentrations of (A) PEI-25 kDa, (B) PEI-750 kDa, or (C) PEI-1000 kDa. Data represent mean ± S.E.M., n = 3–5 [reprinted with permission from Ref. [91], copyright Elsevier (2006)].

In a study conducted recently, the effect of positive-charged polyethylenimine (PEI) on the nasal absorption of LMWH (Enoxaparin) [91]. In this study, the effect of different concentrations of PEIs (0.5%, 0.25% and 0.125%) with different molecular weights (25 kDa, 750 kDa, 1000 kDa) on absorption was demonstrated. The efficacy of PEIs on enhancing nasal bioavailability of LMWH was PEI-1000 kDa > PEI-750 kDa > PEI-25 kDa, respectively. Besides, when PEI-1000 kDa was used at a concentration of 0.25%, the absolute and relative bioavailability of LMWH was increased four-fold when compared to the control formulation (isotonic saline) (Figure 6).

### Other macromolecules

Among other macromolecules whose nasal application has been studied are α-cobrotoxin, exenatide, glucagon, hirudin-2 and octreotide. Below is a brief evaluation of several nasal studies conducted on these molecules.

***α-Cobrotoxin*** is a primary postsynaptic neurotoxic protein consisting of approximately 60-70 amino acid chains, with a molecular weight of 6,951 Da. In treatment it can be applied orally or intravenously, however, these applications have a risk of potential toxicity and adverse effects due to uneven distribution to the whole body and the brain. Therefore, new carrier systems for the molecule have been developed. In a study Li *et al*. [92] prepared microspheres of α-cobrotoxin by using P(CPP:CEFB) and PLGA, which are polyanhydrates. The bioadhesive property of the microspheres was studied on fluorescent microscope, and the presence of P(CPP:CEFB) on microspheres improved the retention time of microspheres on the nasal mucosal surface in rats. A significant increase in the efficacy and time has been noted after i.n. application.

***Exenatide*** is a peptide that contains 39 amino acids, with a molecular weight of 4,186 Da. It has been approved for adjunctive treatment of diabetes type 2. In a recently conducted study, application of exenatide via routes other than the subcutaneous one has been examined. In a study with rats, nasal route has been reported as promising when compared to the subcutaneous route [93].

***Glucagon*** is a peptide containing 29 amino acids, with a molecular weight of 3,482 Da. In a study a nasal powder preparation of glucagon was developed and MCC was used as a nasal absorption enhancer [94]. Following nasal application of mucoadhesive powder prepared at different ratios, a linear relation has been found between MCC concentration and maximum concentration of plasma glucose. In the same study, it has been found that the nasal absorption of glucagons was increased with spray solution containing 1.5% sodium glycolate or 1% sodium caprate.

***Hirudin-2*** is an acidic polypeptide consisting of 65-66 amino acid chains, with a molecular weight of approximately 6,900 Da. It has anticoagulant and antithrombotic activity as one of the most potent inhibitors of thrombin. Recombined hirudin is applied by parenteral routes of intravenous and subcutaneous. In a study, Zhang *et al.* [95] evaluated the systemic absorption and absorption mechanism of recombined hirudin via nasal route. Besides, the effects of penetration enhancers on nasal absorption have been studied in the same study. In conclusion, it has been detected that the penetration enhancers (chitosan (0.5%), hydroxypropyl-β-cyclodextrin (5%), ammonium glycyrrhizinate (1%)) improved nasal absorption when compared to the control. At the end of the *in vitro* transport studies in where rabbit nasal epithelia were used, it has been reported that the absorption mechanism of hirudin can be associated with endocytosis and passive diffusion process.

***Octreotide*** has a molecular weight of 1,019 Da. Octreotide is an octapeptide that pharmacologically mimics natural somatostatin. In order to improve nasal absorption of octreotide, sodium tauro-24,25-dihyrdofusidate has been used as a permeation enhancer [96]. Besides, nasal efficacy of several powder formulations have been evaluated [97]. In this study, MCC, semi-crystallized cellulose, hydroxyethyl starch, cross-linked dextran (Sephadex^®^ G25), microcrystalline chitosan, pectin and alginic acid were used as polymer. First of all, the Ca^2+^-binding capacity of these polymers was evaluated, and Sephadex^®^ G25 and alginic acid which have highest Ca^2+^-binding capacity. Therefore, the highest bioavailability among the nasally applied powders to rats has been achieved with Sephadex^®^ G25 and alginic acid. In conclusion, this study has demonstrated a correlation between the Ca^2+^-binding property of nasal carriers and the potential of peptides to improve nasal absorption.

### Vaccines

Today, there are approximately 26 types of vaccines used in different infectious diseases. However, these common vaccines have the disadvantages of mandatory use as an injection, side effects and stability problems (need to be stored in refrigerator). Besides, most of the newly introduced proteinic vaccines are weakly immunogenic. Therefore, development of new types of vaccines is of critical importance. Recently, mucosal immunization has been offered as an alternative to the currently used vaccines in treatment. Besides, as the mucosal areas are the entrance gates of 80% of all infections, mucosal immunization is provided in the area where the pathogen enters the body and the infection is blocked without spreading. Mucosal vaccination has many advantages when compared to the conventional vaccination [98,99,100,101], which can be listed as follows: mucosal vaccines are easy to apply; large numbers of people can be vaccinated at lower costs; there is no staff requirement for vaccination; and it prevents the possibility of cross-contamination due to insufficiently sterilized needles or insufficient hygiene. Besides, mucosal vaccination offers both mucosal and humoral immunity. Vaccination efficacy can be improved among the elderly and the infants in certain cases such as flu. Mucosal immune system is functionally different than the systemic immune system. The nasal route is one of the mucosal immunization routes and is one of the most frequently tried and promising routes along with the oral route. The first nasal vaccine was the nasal influenza vaccine which was introduced to the European market in 2001. However, it was later withdrawn from the market due to potential toxicity problems. The second product (FluMist^®^ MedImmune, Inc.) which was put on the market in 2003 was another influenza vaccine indicated for the active immunisation of individuals 2-49 years of age against influenza disease caused by influenza virus types A and B contained in the vaccine. This product is given as one or two doses over the influenza season via a syringe sprayer.

The advantages of nasal route in mucosal vaccination are a) the area is the first contact area of the inhaled antigen, b) the nasal passages are rich in lymphoid tissue (nasal asssociated lymphoid tissue-NALT), c) low abrasive effect of low acid pH and low ratio of proteolitic enzymes for the antigen, d) easier uptake of particles via nasal cavity when compared to intestinal uptake, e) availability of both mucosal (IgA) and systemic (IgG) response via this route, and f) provision of immune response to the antigen even when applied in low doses [99,100].

However, nasal vaccines have some several limitations, including mucociliary clearance and the inefficient uptake of soluble antigens. Therefore, mucosal adjuvants are required to achieve an effective nasal immunization. As mucosal adjuvants, bacterial toxins, such as cholera toxin B subunit A and mutant *Escherichia coli* heat-labile toxin, which have weak toxicity, as well as recombinant living organisms (bacterial/viral) and biodegradable nano-/micro-particles are used [102]. 

There are numerous studies on nano- and micro-particles to develop nasal vaccines [103,104,105,106]. The aim is to develop single-dose vaccines with long-enduring effect due to numerous factors such as hydrophobicity of the particulate system used, surface charge and particle size [102,103]. Examples of the studies dealing with these factors are provided below.

In a study, Singh *et al*. [107] have evaluated the effect of hydrophobicity of the polymer used in preparation of particulate system on the Caco-2 cell uptake of the particulate system. Moreover, they looked into the immune reponse after nasal application of these nanoparticles which contain diphtheria toxoid. Poly-ε-caprolacton nanoparticles induced significantly higher serum diphtheria toxoid specific IgG antibody responses than PLGA. The positive correlation between hydrophobicity of the nanoparticles and serum diphtheria toxoid specific IgG antibody response was observed after intranasal administration of the nanoparticles.

**Figure 7:**
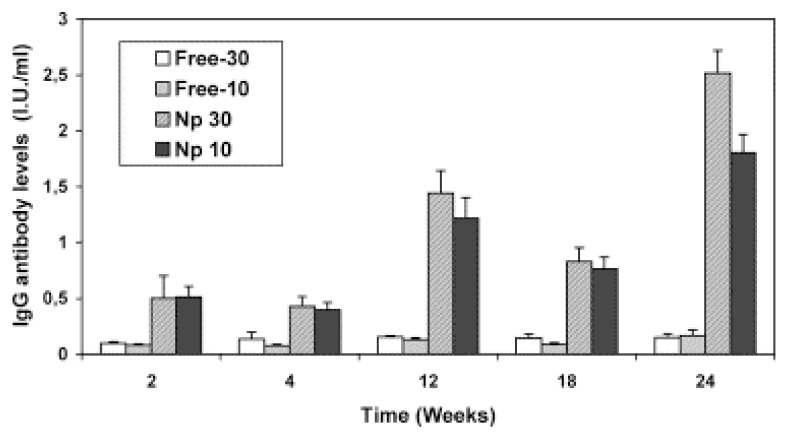
IgG antibody levels after i.n. administration of two doses of antigen (10 and 30 μg), encapsulated in chitosan nanoparticles (70 kDa) or in solution in mice (geometric mean ± SEM) [reprinted with permission from Ref. [109], copyright Elsevier (2004)].

The surface charge of the particulate system is another parameter affecting the performance of vaccines. M-cell and epithelium cells are negatively charged. It is supposed that the positively charged groups on particles interact with the negatively charged cell membranes and achieve a high antigen uptake. Chitosan is a frequently used natural polymer in nasal vaccine systems due to its positive charge and ability to open tight-junctions [108]. In a study where tetanus toxoid-loaded nanoparticles were prepared with chitosan, nanoparticles were nasally applied to mice [109]. Tetanus toxoid-loaded nanoparticles (Np 10 and Np 30) have achived a high serum IgG level when compared to free antigen (Free-10 and Free-30) (Figure 7). 

The M-cells in the NALT uptakes the smaller particles (particularly the nanoparticles) more rapidly, and therefore the vaccine studies with particulate systems focus rather on development of smaller particulate systems. In a study, Tetanus toxoid-loaded particles were prepared at varying sizes of 100 nm, 500 nm and 1500 nm. Following their nasal application, the particles of 100 nm and 500 nm achieved significantly higher serum IgG and IgA responses than those of the particles of 1500 nm [110].

As mentioned above, there are numerous studies on the nasal application of vaccines particularly because nasal route is an attractive route for vaccines, and it may be the subject of another chapter, therefore, the vaccines have been mentioned only briefly in this compilation.

## Conclusions

The nasal route has become one of the most studied routes non-invasive routes because it offers a vast surface area, a thin membrane structure and a rich vascularity. The nasal epithelia barrier constitutes an obstacle for the drugs larger than 1,000 Da. Therefore, it is difficult for macromolecules, particularly those which are hydrophilic, to pass through the nasal mucosa. Moreover, the enzymes present in the nasal mucosal membrane constitute a problem for the stability of macromolecules with a peptide and protein structure. Numerous strategies have been developed to improve the passage of macromolecules through the nasal mucosa. Among these, the most studied one is addition to the formulation of permeation enhancers and development of polymer-based particulate systems. Enhancers can improve the passage of macromolecules through nasal mucosa via different mechanisms. Particulate systems, however, extend the retention time of the drug in the mucosa and open the tight junctions between epithelial cells to improve drug absorption. Besides, the drugs with protein and peptide structure applied with particulate systems are protected against the enzymatic activity in the nasal mucosa. Nasal application of vaccines has gained importance in the recent years. Particularly the nasal vaccines applied with particulate systems achieve both mucosal and systemic immune response. Therefore, nasal application of vaccines with these systems is a promising method. These vaccines will provide better protection against infection, and possibility of a wider and safer use in Third World countries. It is expected that novel formulations with enhancers and/or particulate systems of macromolecular drugs will be on the shelves of pharmacies in the near future.

## Figures and Tables

**Table 1:** Commercially available macromolecular drugs applied via nasal route.

Drugs	Molecular weight (Da)	Formulation	Commercial name	Company	FDA approval date
Desmopressin acetate	1183	Solution, Spray	Minirin^®^	Sanofi-Aventis	1978
Salmon calcitonin	3432	Solution, Spray	Miacalcin^®^	Novartis	1995
Buserelin acetate*	1239	Solution, Spray	Suprefact^®^	Sanofi-Aventis	-
Nafarelin acetate	1321	Solution, Spray	Synarel^®^	Pfizer	1990
Oxytocin	1007	Solution, Spray	Syntocinon^®^	Novartis	1995
Cyanocobalamine	1355	Gel	Nascobal^®^	Par Pharm Co.	1996
Cyanocobalamine	1355	Solution, Spray	Nascobal^®^	Par Pharm Co.	2005

* Approved in Canada.

**Table 2:** Studies on nasal insulin formulations.

Polymers/Others	Delivery system	Enhancer	Animal model	Results	Ref.
Degradable starch	Microspheres	LFC	Sheep	While the relative bioavailability of insulin from microspheres was 10.7%, addition of enhancer to the formulation, bioavailability of insulin was increased to 31.5%.	[45]
Soluble starch	Powder and Microspheres	-	Rats	A comparison between microspheres and starch powders (mw 11000 and 25000) indicated that the insoluble starch of mw 25000 and the microspheres reduced the plasma glucose level to the same extent. Besides water soluble starch powder (mw 11000) did not change the plasma glucose level.	[46]
Crosslinked starch and Dextran	Microspheres	Epichlorohydrin	Rats	The effect on the glucose level of insulin from starch and dextran microspheres was rapid and maximum decrease in plasma glucose level was achieved in 30-40 minutes. The effect of starch microspheres was found more efficient than that of dextran microspheres to decrease blood glucose level.	[47]
Starch- Carbopol^®^ 974P and maltodextrin -Carbopol^®^ 974P	Freeze-dried powder	-	Rabbits	The nasal bioavailability achieved with the application of Starch-Carbopol^®^ 974P powder was significantly higher than that of the maltodextrin-Carbopol^®^ 974P mixtures.	[48]
Starch	Microspheres	Bile salt derivatives (LFC, GDC, STDF)	Sheep	Bioadhesive starch microspheres have improved transport of insulin across nasal membrane in the presence of absorption enhancers. Addition of enhancer to the microspheres has increased insulin absorption than that of absorption enhancer in solution.	[49]
Amioca^®^ starch and Carbopol^®^ 974P	Powder	-	Rabbits	Following nasal single-dose application of a physical mixture of Amioca^®^ starch and Carbopol^®^ 974P (9/1) the bioavailability of insulin has been found to be more than 10%.	[50]
Crosslinked starch	Nanoparticles	SGC, LFC	Rats	A rapid hypoglycemic effect has been observed with nasal application of nanoparticles. It has been emphasized that the release of insulin from nanoparticles can be modified by adjusting the degree of cross-linking. The release rate of insulin has significantly increased with combination of permeation enhancers and nanoparticles.	[52]
Dextran	Microspheres	-	Rats	Microspheres with insulin on the surface were more effective in promoting insulin absorption than those with insulin distributed within the dextran matrix.	[53]
Anionic resin (SPS), nonionic resins (PAE, SDBC) and cationic resin (CA)	Powder	-	Rabbits	Nasal administration of insulin mixed with anionic resin caused a rapid increase of the plasma insulin level, while nasal administration of insulin alone caused little increase. Nonionic resin (SDBC) showed similar enhancement in nasal insulin absorption in contrast, the other nonionic resin and cationic resin did not improve insülin absorption.	[54]
Hyaluronic acid ester	Microspheres	-	Sheep	Average relative bioavailability of insulin from microspheres was calculated as 11% when compared with insulin administered by subcutaneous route.	[55]
Chitosan	Nanoparticles	-	Rabbits	The freeze-dried formulation of insulin-loaded chitosan nanoparticles has led to a greater decrease in plasma glucose level when compared to the insulin chitosan solution.	[56]
Cross linked chitosan	Nanoparticles	-	Rats	Microspheres containing chitosan and ascorbyl palmitate caused a 67% reduction of blood glucose compared to intravenous route and absolute bioavailability of insulin was found as 44%.	[57]
Thiolated chitosan	Nanoparticles	-	Rats	Insulin-loaded thiolated chitosan microspheres let to more than 1.5-fold higher bioavailability and more than 7-fold higher pharmacological efficacy than unmodified chitosan microspheres.	[58]
Chitosan	Nanoparticles	CM-β-CD	-	The fast release of insulin from chitosan/CM-β-CD nanoparticles was observed (84-97% insulin within 15 min.).	[59]
Chitosan	Nanoparticles	-	Rats	Nanoparticles containing insulin have increased the pharmacodynamic activity of the drug. The synthesis of gold nanoparticles prepared by using chitosan has used a new method, and therefore, the surface properties of chitosan were improved for binding of biomolecules.	[60]
Chitosan	Nanoparticles	NAC	Rats	Nasal administration of chitosan-NAC nanoparticles increased the insulin absorption compare to unmodified chitosan nanoparticles and control insulin solution.	[61]
Chitosan	Nanoparticles	SBE-β-CD and CM-β-CD	Rabbits	The nanoparticles have reversibly increased the transepithel resistance of the cells and increased the membrane permeability in in-vitro cell culture studies. Nasal application of fluorescence-loaded nanoparticles to rats has proved their ability to pass through nasal mucosa. In conclusion, insulin-loaded nanoparticles have decreased the plasma glucose level (more than 35% reduction).	[62]
Aminated gelatin	Microspheres	-	Rats	Aminated gelatin microspheres have significantly increased the nasal absorption of insulin when administered in dry formulation but no significant hypoglycemic effect was observed when given as a suspension.	[63]

LFC = Lysophosphatidylcholine; GDC= Glycodeoxychlote; STDF = Sodium taurodihydroxyfusidate.

SPS = Sodium polystrene sulphonate; PAE = Polyacrylester; SDBC = Styrene-diviniylbenzene copolymer; SGC = Sodium glycocolate.

CA = Cholestramine; CM-β-CD = Carboxymethyl-β-cyclodextrin; NAC = N-acetyl-L-Cysteine; SBE-β-CD = Sulfobutylether-β-cyclodextrin.

**Table 3:** Studies on the nasal heparin formulations.

Type of heparin	Formulations	Penetration enhancers	Animal model / Human	Results	Ref.
UFH	Aqueous solution	-	Human	Heparin showed the protection with respect to nasal allergic challenge.	[84]
UFH	Aqueous solution	-	Human	Nasal heparin showed a protective role against AMP provocation by inhibition of mast cell activation.	[85]
UFH	Poly(L-lactic acid) microspheres	-	Rats	Nasal application of poly (L-lactic acid)-heparin microspheres had a relative bioavailability of 143% (vs nasal heparin solution).	[86]
UFH	Chitosan nanoparticles	carboxymethyl-β-cyclodextrin	-	Heparin was released slowly from chitosan/cyclodextrin nanoparticles (8.3-9.1% heparin within 8 h).	[59]
Enoxaparin Dalteparin UFH	Aqueous solution	Tetradecymaltoside	Rats	The addition of tetradecymaltoside into nose drops formulations containing LMWH has let to in a significant increase in the Cmax and AUC of anti-factor Xa activity compare to LMWH in saline. But the addition of tetradecymaltoside into formulations containing UFH has let to much smaller increase in the Cmax and AUC of anti factor Xa activity.	[87]
LMWH	Aqueous solution	Dimethyl-β-cyclodextrin	Rats	Dimethyl-β-cyclodextrin was found the most effective enhancer for the absorption of LMWH.	[88]
LMWH (Enoxaparin)	Aqueous solution	Alkylmaltosides	Rats	Alkylmaltosides improved the nasal absorption of LMWH without causing an irreversible damage in nasal mucosa. When the alkyl chains of maltosides were increased from 8 to 14 carbons, absolute and relative bioavailability of Enoxaparin were increased by two-fold.	[89]
LMWH (Enoxaparin)	Aqueous solution	Alkonoylsucroses	Rats	The enhancers increased the bioavailability of LMWH when compared to saline solution. The potency of these enhancers was dependent on their hydrophobic chain lengths.	[90]

UFH = Unfractionated heparin; LMWH = Low molecular weight heparin.
